# Electric tuning of magnetization dynamics and electric field-induced negative magnetic permeability in nanoscale composite multiferroics

**DOI:** 10.1038/srep11111

**Published:** 2015-06-09

**Authors:** Chenglong Jia, Fenglong Wang, Changjun Jiang, Jamal Berakdar, Desheng Xue

**Affiliations:** 1 Key Laboratory for Magnetism and Magnetic Materials of MOE, Lanzhou University, Lanzhou 730000, China; 2 Institut für Physik, Martin-Luther Universität Halle-Wittenberg, 06099 Halle (Saale), Germany

## Abstract

Steering magnetism by electric fields upon interfacing ferromagnetic (FM) and ferroelectric (FE) materials to achieve an emergent multiferroic response bears a great potential for nano-scale devices with novel functionalities. FM/FE heterostructures allow, for instance, the electrical manipulation of magnetic anisotropy via interfacial magnetoelectric (ME) couplings. A charge-mediated ME effect is believed to be generally weak and active in only a few angstroms. Here we present an experimental evidence uncovering a new magnon-driven, strong ME effect acting on the nanometer range. For Co_92_Zr_8_ (20 nm) film deposited on ferroelectric PMN-PT we show via ferromagnetic resonance (FMR) that this type of linear ME allows for electrical control of simultaneously the magnetization precession *and* its damping, both of which are key elements for magnetic switching and spintronics. The experiments unravel further an electric-field-induced negative magnetic permeability effect.

Magnetic-based information processing as realized for instance in memory devices and sensors relies on switching the magnetization (**M**) between equilibrium states. Such a process evolves in general through a damped precession. The magnetic precession is directly governed by the effective magnetic field **H**_eff_ whereas the precessional damping is dependent on *α*, the dimensionless Gilbert damping parameter^1^. Engineering both, **H**_eff_ *and α* is thus of a key importance for a reliable and swift magnetic switching^2^,^3^. In a coupled FE/FM system **H**_eff_ is augmented by an additional interfacial ME interaction^3^,^4^,^5^,^6^,^7^,^8^,^9^,^10^,^11^ and hence becomes dependent on the ferroelectric polarization that is inherently controllable by electrical means. This opens the door for the electric control of **M**, as shown in recent studies on voltage-controlled magnetic anisotropy via FMR excitation in appropriately synthesized FM/FE heterostructures^12^,^13^,^14^,^15^. The damping is a nonlinear spin relaxation of precession due to the combined effects of intrinsic impurity scattering^16^,^17^ and spin-orbit interactions^18^,^19^, as well as extrinsic nonlocal spin pumping^20^,^21^,^22^, and is vital for achieving high-speed magnetic functionality. It is however challenging to manipulate damping, particularly by electrical means, even though possible magnetic control of damping has been observed in some left-handed metamaterials^23^,^24^,^25^ close to FMR frequency^26^,^27^,^28^.

A direct gate-voltage control of magnetic permeability is still an open question despite its key role in ME devices and metamaterials with negative refraction index. A way to achieve this is offered by a recently predicted mechanism of ME coupling for FM/FE^29^; according to which in FM a spiral magnetic ordering builds up at FM/FE interface with a nanometer range determined by spin diffusion length. This non-collinear ordering is coupled to the FE polarization (a brief summary of mathematical details is included in the supplementary materials to this paper). Since spiral order entails a spin orbital coupling^30^ we expect thus a gate-voltage dependent influence on damping (via coupling to polarization). Indeed, we report here FMR experiments evidencing that both the effective magnetic field and Gilbert precessional damping are directly controllable in Co_92_Zr_8_(CoZr)/Pb(Mg_1/3_Nb_2/3_)_0.7_Ti_0.3_O_3_(PMN-PT) heterostructure by means of a gate voltage. Furthermore, we find that magnetic excitations pumped by the external electric field by virtue of the magnetoelectric may result in electric field-induced negative imaginary part of magnetic susceptibility (permeability) at room temperature.

## Results

Several ME coupling mechanisms in composite FM/FE heterostructures were envisaged, in particular ME-effect driven by spin-polarized screening, strain, or/and exchange bias^3^,^4^,^5^,^6^,^7^,^11^,^31^. A detailed analysis show, however, that the latter two indirect ME interactions: the interfacial strain-mediated and exchange-mediated couplings, contribute with an additional magnetic-anisotropy type term to the FM free energy *F*, i.e., they alter *only* the effective magnetic field by **H**_eff_ = −*δF*/*δ***M** (which governs directly the precessional motion) but they have no direct influence on the damping parameter *α* of the magnetization evolution **M**(*t*). This is readily inferred from the phenomenological Landau-Lifshitz-Gilbert (LLG) equation 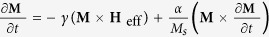 (here *γ* is the gyromagnetic ratio and *M*_*s*_ is the saturation magnetization). This also applies to screening of FE polarization via interfacial charge accumulation. What about spin-driven ME coupling? As shown recently, when a FM is brought in contact with a FE a non-collinear spin density **s**(**r**, *t*) develops in the FM interface on the scale of the spin diffusion length λ_*m*_ in response to the adjacent FE polarization^29^. Ensuring electric neutrality one finds: *(i)* A linear ME coupling and thus an effective gate-controlled magnetic field. *(ii)* An induced spin (orbital) torque resulting in an electrically tunable effective magnetic damping. Furthermore, considering that 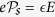 with *e* and 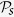 being the electron charge and the surface electrostatic screening charge density respectively, the induced spin density **s**(**r**, *t*) is linearly determined by the applied electric field *E*^32^,^33^,^34^,^35^,^36^ and the dielectric permittivity *ε* (in the unit of the vacuum permittivity ε_0_) at the FM/FE interface which can be significantly enhanced up to several orders (compared with ε ~ 1 at the interface of the bulk FM metal). On the other hand, due to the large spin-diffusion length in FM metals^37^, ME effects may have substantial influence on FM films with thickness over tens of nanometers. Having these properties in mind we have chosen polycrystalline Co_92_Zr_8_ (20 nm) layer with an intrinsic damping *α* = 0.008 and Pb(Mg_1/3_Nb_2/3_)_0.7_Ti_0.3_O_3_ with *ε* ≈ 4–6 × 10^3^ as our candidates for FM and FE subsystem, respectively. It should be noted that similar results are observed for CoZr film with a thickness equal to 10 nm.

**Figure 1:**
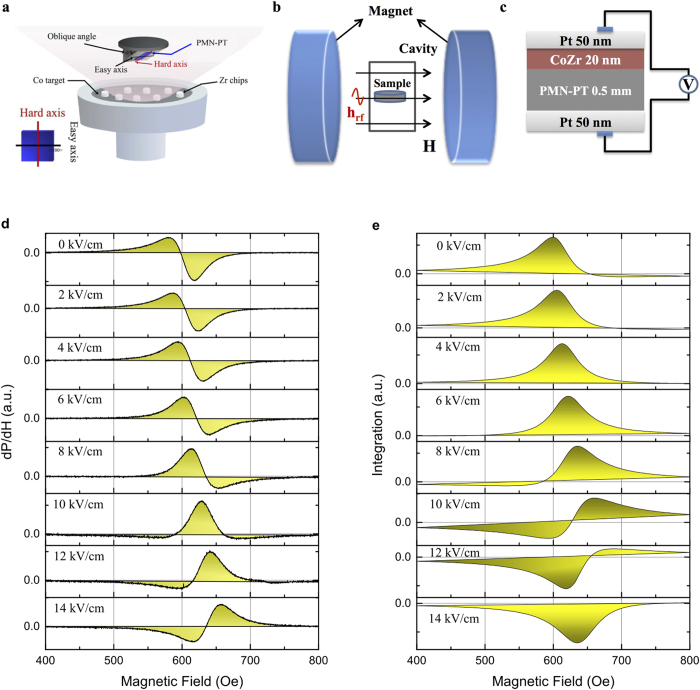
Observation of the electric-field control of magnetic dynamics. Schematic of composition system (**a**) measurements of FMR spectroscopy (**b**) and CoZr/PMN-PT heterostructures (**c**). The FMR spectra, obtained by fixed frequency *ω* = 8.969 GHz and by sweeping the external magnetic field *H* at room temperature, under different applied electric field (**d**) and replotted in integrated intensity (**e**). Symbols: experiment, gray lines: fits to the asymmetric Lorentz function.

A sketch of the sample and the measurement configuration is shown in Fig. 1a–c. The CoZr thin films were prepared on one-side-polish (011)-oriented PMN-PT single-crystal substrates at room temperature by radio frequency magnetron sputtering. A vibrating sample magnetometer (VSM) measurements demonstrated an electric field control of magnetic hysteresis loops with an in-plane uniaxial magnetic anisotropy (cf. Fig. s1 in the Supplementary). In Fig. 1d, the dynamic magnetoelectric response was investigated by using a more direct and sensitive FMR spectroscopy^38^ at room temperature under different gate voltages. In the bias range 0–14 kV/cm, resonance spectra with dispersion structure were observed. The form of the FMR line shape, however, differs quantitatively in low and high external electric fields which becomes increasingly obvious after integrating the signals over the applied static magnetic field (cf. Fig. 1e). A clear change in the sign of the integrated Lorentzian lineshape is observed by comparing the profiles in the two limit cases with applied electric field 0 kV/cm and 14 kV/cm. This is insofar important as the microwave power, *P*(*H*), absorbed by the CoZr film in our experimental geometry reads *P (H) *= *ωℑχ_z_* |*h*|^2^ /2 with *h* being the amplitude of the applied rf driving field with frequency *ω* = 8.969 GHz and *ℑχ*_*z*_ is the imaginary part of dynamic magnetic susceptibility. Thus, the experiment unravels the emergence of *positive-to-negative* transition of *ℑχ*_*z*_ in natural FM metal by applying external electric field for coupled FM/FE structures. We note, that negative *ℑχ*_*z*_ does not mean that the system is dissipating negative energy, since in the presence of the external electric field (and the magnetoelectric coupling) the energy is not conserved. It is rather so, that excitations triggered by the electric field are transferred to the spin system via the magnetoelectric coupling and this shows up in the FMR, effectively as negative *ℑχ*_*z*_. This novel observation we expect to have important implications for future applications of composite FM/FE systems to optical applications.

**Figure 2:**
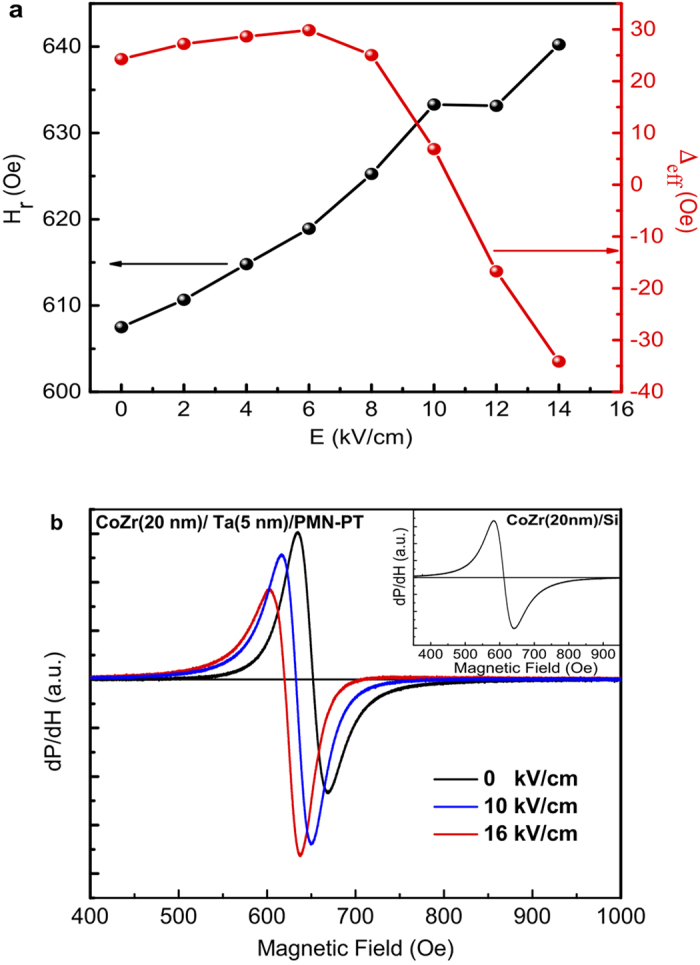
(**a**) Effective resonance magnetic field *H*_*r*_ and line broadening Δ_eff_ obtained by fitting the integrated experimental data to an asymmetric Lorentzian lineshape (solid dots). (**b**) FMR spectra of CoZr/Ta/PMN-PT heterostructure under different external electric fields. The inset shows a symmetric spectrum obtained in the CoZr/Si composite structure.

For an insight into the electrical tuning of magnetic dynamics, we study the resonance spectra based on the phenomenological LLG equation, which has been widely used for interpreting and predicting vast experimental results of magnetic structures (See Ref. 39 and the supplementary). We find


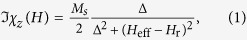


where *H*_r_ is the resonance field, Δ characterizes the half-maximum linewidth, connected to the relaxation processes by 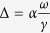. In principle, the integrated absorption lineshape is almost perfectly Lorentzian as Δ 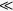 *H*_*r*_,^40^ which is fully fulfilled by our absorption experiments. The same measurement performed with a CoZr thin film of equivalent thickness on top of Si substrate also possesses a perfectly symmetric resonance lineshape in all applied bias ranges (the inset in Fig. 2b). Whereas, as for small damping and if the magnetization **M** being parallel to the externally applied magnetic field, a non-zero phase shift would be expected and the line shape of FMR spectrum does not exclusively correspond to imaginary magnetic susceptibility, as follows from LLG equation, but represents a mixture of imaginary and the real part of the susceptibility^41^. Given the asymmetric nature of the real part of the dynamic susceptibility as inferred from LLG, we conclude that the asymmetric profile at moderate electric fields in Fig. 1e is a mixture effect. Solid lines in Fig. 1e show fits with an asymmetric Lorentzian function. The derived values of the resonance field *H*_*r*_ and effective inhomogeneous line broadening, Δ_eff_ = Δcos *ϕ* with *ϕ* being the mixture phase between the real and imaginary part of the susceptibility, are shown in Fig. 2a; the electric-field-dependence of *H*_*r*_ is almost linear. We note that together with an effective dielectric permittivity *ε* ≈ 10^3^ at the interface, the linear fit of *E* to *H*_*r*_ indicates an interfacial linear magnetoelectric interaction with a strong coupling strength that amounts to 0.32 s/F, in good agreement with our theoretical prediction^29^,^42^,^43^ and the experimental estimation in Co/BaTiO_3_ thin films^13^. Furthermore, the linear growth of *H*_*r*_ with *E* implies an increasing negative surface spin density, which will give rise to a decrease of the effective damping, and more detectable, a sign transition in small *α* at the critical electric field. Taking the odd-parity of the imaginary part of the magnetic susceptibly and its relation to damping *α*^39^, we expect a phase transition close to the frequency of FMR, which is in line with the experimental observations. The nonmonotonic behavior of the effective Δ_eff_ may be due to a nonlinear interplay of the higher order effects with nonlocal damping^22^, originating from the dissipative flow of nonequilibrium intralayer spin currents within the interface.

Experimentally, several multiferroic heterostructures based on (011)PMN-PT, such as Fe_3_O_4_/PMN-PT^44^, Ni_0.79_Fe_0.21_/PMN-PT^14^, and Ni_0.46_Zn_0.54_Fe_2_O_4_/PMN-PT^45^ were realized, the magnetic properties are found to be co-mediated by the interfacial strain- and screening-driven ME interaction due to the piezoelectricity of PMN-PT. To confirm the absence of stress-strain effect on the damping in our measurements, a 5 nm Ta layer was inserted between the CoZr and PMN-PT substrate to have a CoZr/Ta/PMN-PT heterostructure. Due to the isolation by the metallic (non-magnetic) Ta film with small dielectric permittivity, no considerable screening charge is expected on CoZr layer. A strain-mediated ME coupling transferred through the Ta thin layer remains as the main factor that influences the magnetization dynamics. Its consequence is a shift of the resonance field only, which is consistent with the experimental results (Fig. 2b). No noticeable changes, let alone a reversal is observed in *ℑχ*_*z*_ up to 16 kV/cm. To further clarify the interfacial strain effect on CoZr/PMN-PT heterostructure itself, we investigated the angular dependence of the resonance field for CoZr/PMN-PT film when varying the applied static magnetic field in-plane. Typically, the influence of the stress-strain effect on the magnetic anisotropy is more pronounced than the surface anisotropy stemming from the screening effect at the FM/FE interface. Inspecting the values under *E* = 0 kV/cm and those for *E* = 8 kV/cm, the FMR effective magnetic field *H*_*r*_ should possesses a large amplitude modification, as reported in the previous experiment on PMN-PT-based heterostructures^14^,^45^. However, under the present measurement conditions, *the change* concerning the amplitude of *H*_*r*_ at each angle, are small (cf. Fig. s2 in the Supplementary). Its angular dependence is found to be well described by an in-plane uniaxial anisotropy, and the phase-shift of *H*_*r*_ under different applied external electric fields can be understood as an angle mismatch of the easy axis of the uniaxial anisotropy with respect to the [100]-direction. Such a small contribution of the strain-mediated ME interaction to the magnetic dynamics hints on the polycrystalline nature of our CoZr film, the average on the randomly oriented grains may suppress possible magnetostrictive effects, similar to polycrystalline Co/BaTiO_3_ heterostructure in Ref.13.

## Discussion

The direct electric control of magnetic dynamics reported here offers a qualitatively new way to manipulate multiferroic devices with fast low-power heterogeneous read/write capability through the interfacial ME interaction. The static linear coupling manifests itself as an effective magnetic field and the dynamic coupling possesses substantial influence on magnetic damping in the thin FM film. A further interesting point is that a fully electric-field control of magnetic permeability can be realized in natural FM metals in contact with FE by external electric field at room temperature. Indeed, our recent direct permeability measurements of polycrystalline CoZr/PMN-PT and Co/PMN-PT show the emergence of positive-to-negative transition in both real and imaginary parts of magnetic permeability. Given that the dielectric permittivity of metal is negative below plasma frequent, the electric tuning of magnetic permeability which opens new perspectives to envisage spin-wave amplification by stimulated emission of radiation device^46^.

## Methods

The base pressure was 2.5 × 10^−5^ Pa, and a 0.2 Pa pressure of Argon was used in the sputtering. Co target with regularly placed Zr chips, which was 70 mm in diameter and 3 mm in thickness, was used to deposit the CoZr layer. The composition of the deposited magnetic layer was adjusted by controlling the number of the Zr chips, as shown in Fig. 1a in the main text. The film was deposited at oblique angle 20^°^ without any applied field, which induced in-plane uniaxial anisotropy. Pt layers were sputtered on both the top and bottom sides of the FM/FE structure as electrodes. Cu wires were connected to the electrodes and dc voltage was applied to the PMN-PT along (011) with an electrometer. The magnetic properties of the samples were measured by a vibrating sample magnetometer. An in-plane uniaxial magnetic anisotropy is evident by a rectangle easy-axis hysteresis loop with the remanence ration *M*_*r*_/*M*_*s*_ ≈ 1. Further details of electric field induced changes of the static magnetic properties are given in the supplementary material to this paper. In-plane FMR measurements were performed in a JEOL, JES-FA 300 (X-band at *ω* = 8.969 GHz with the power 1 mW) spectrometer at room temperature. As shown in Fig. 1b of the main text, the FMR setup. The electromagnet produces the static in-plane magnetic field. A cylindrical cavity resonator is placed at the center of the electromagnet. The microwave unit connected with the cavity resonator by waveguide generates microwave (the rf magnetic field is normal to the FM/FE films) and detects the reflection. As for the influence of the current, no current was injected into the ferromagnetic layer in the experiment. The leakage current during the measurements is below nA, which is much smaller than any other relevant effect in the system and also ways below the required current density for a current induced torque acting on the magnetization.

## Additional Information

**How to cite this article**: Jia, C. *et al*. Electric tuning of magnetization dynamics and electric field-induced negative magnetic permeability in nanoscale composite multiferroics. *Sci. Rep*. **5**, 11111; doi: 10.1038/srep11111 (2015).

## Supplementary Material

Supplementary Information
