# Supplementary material for: Electric tuning of magnetization dynamics and electric field-induced negative magnetic permeability in nanoscale composite multiferroics
Source: Sci Rep. 2015 Jun 9;5:11111. doi: 10.1038/srep11111 (PMC4460868; doi:10.1038/srep11111)
Supplement: Supplementary Information [file srep11111-s1.pdf]

Supporting Information: Electric tuning of magnetization dynamics and electric field-induced negative magnetic permeability in nanoscale composite multiferroics

Chenglong Jia[1,2], Fenglong Wang[1], Changjun Jiang[1], Jamal Berakdar[2], and Desheng Xue[1]

[1]*Key Laboratory for Magnetism and Magnetic Materials of the MOE, Lanzhou University, Lanzhou 730000, China*

[2] *Institut für Physik, Martin-Luther Universität Halle-Wittenberg, 06099 Halle (Saale), Germany*

INTERFACE MAGNETOELECTRIC EFFECT

Here we give a summary of the main implications following from an interfacial magneto-electric (ME) coupling based on the formation of a spiral magnetic order that is coupled to the polarization in a ferroelectric (FE) interfaced with ferromagnet FM [1]. In a composite FM/FE multiferroics a spin polarized nonequilibrium density $\hat{\mathbf{s}} = \sum_{\sigma\sigma'} \psi^\dagger_\sigma(\mathbf{r})\hat{\boldsymbol{\sigma}}_{\sigma\sigma'}\psi_\sigma(\mathbf{r})$ develops in FM interface due to the surface FE polarization. $\psi^\dagger_\sigma$ and ψ_σ are respectively the electron creation and annihilation operators, satisfying the anti-commutation relation $\{\psi_\sigma(\mathbf{r}), \psi^\dagger_{\sigma'}(\mathbf{r}')\} = \delta(\mathbf{r}-\mathbf{r}')\delta_{\sigma\sigma'}$. In the mean-field approximation, $\hat{\mathbf{s}}$ interacts with localized spins $\mathbf{S}$ via s-d exchange interaction [2], $H_{sd} = J_{ex}\hat{\mathbf{s}}\cdot\mathbf{e}_{\mathrm{M}_\parallel}$ with $\mathbf{e}_{\mathrm{M}_\parallel} = \mathbf{M}/M_s$ and the classical magnetization $\mathbf{M} = -\frac{g\mu_B}{a^3}\mathbf{S}$, where μ_B, g and a are the Bohr magneton, g-factor, and lattice constant, respectively. Together with the kinetic energy and the electrostatic potential $V(\mathbf{r})\hat{n}(\mathbf{r})$ with $\hat{n}(\mathbf{r}) = (-e)\sum_\sigma \psi^\dagger_\sigma(\mathbf{r})\psi_\sigma(\mathbf{r})$ being the charge density operator, the total Hamiltonian for non-interacting surface electrons reads

$$H = \frac{\hbar^2}{2m}\sum_\sigma\int d\mathbf{r}\boldsymbol{\nabla}\psi^\dagger_\sigma(\mathbf{r})\cdot\boldsymbol{\nabla}\psi_\sigma(\mathbf{r}) + \int d\mathbf{r}\left[V(\mathbf{r})\hat{n}(\mathbf{r}) + J_{ex}\hat{\mathbf{s}}\cdot\mathbf{e}_{\mathrm{M}_\parallel}\right]. \quad (1)$$

Upon considering the dynamics of the spin density operator $\hat{\mathbf{s}}$ based on the Heisenberg equation, $\frac{d\hat{\mathbf{S}}}{dt} = \frac{1}{i\hbar}[\hat{\mathbf{s}}, H]$, we obtain a Bloch equation for the spin density $\mathbf{s} = \langle\hat{\mathbf{s}}\rangle$ in the semiclassical approach [3],

$$\frac{d\mathbf{s}}{dt} + \boldsymbol{\nabla}\cdot\mathcal{J} = -\frac{1}{\tau_{ex}}\mathbf{s}\times\mathbf{e}_{\mathrm{M}_\parallel} - \frac{\mathbf{s}}{\tau_{sf}} \quad (2)$$

where $\mathcal{J} = \frac{\hbar^2}{2m}\langle\Im[\psi^\dagger\hat{\boldsymbol{\sigma}}\bigotimes\boldsymbol{\nabla}\psi]\rangle$ is the spin current density with nonequilibrium surface electron charge buildup. No steady charge currents occur in the present FM/FE setup. The spin current is thus related only to the nonequilibrium spin density $\mathbf{s}$ along the interface normal direction z via $\mathcal{J} = -D_0\boldsymbol{\nabla}_z\mathbf{s}$, where D_0 is the diffusion constant. $\tau_{ex} = \hbar/J_{ex}$, and τ_{sf} being the spin-flip relaxation time due to scattering with impurities, electrons, and phonons, etc. In general, the spin polarization η of electron density in transition FM metals is less than 1 within the Stoner mean-field theory [4], it is therefore instructive to separate the induced spin density into two parts,

$$\mathbf{s}(\mathbf{r},t) = \mathbf{s}_\parallel(\mathbf{r},t) + \mathbf{s}_\perp(\mathbf{r},t) \quad (3)$$

where $\mathbf{s}_\parallel$ represents the spin density whose direction follows approximately the intrinsic magnetization $\mathbf{M}$ due to local exchange couplings at an instantaneous time t, i.e., $\mathbf{s}_\parallel = s_\parallel \mathbf{e}_{\mathrm{M}_\parallel}$. $\mathbf{s}_\perp$ describes the transverse deviation from $\mathbf{M}$. Then eq. (2) can be rewritten as

$$\frac{\partial s_\parallel}{\partial t}\mathbf{e}_{\mathrm{M}_\parallel} + s_\parallel \frac{\partial \mathbf{e}_{\mathrm{M}_\parallel}}{\partial t} + \frac{\partial \mathbf{s}_\perp}{\partial t} - D_0 \boldsymbol{\nabla}_z^2 \mathbf{s}_\parallel - D_0 \boldsymbol{\nabla}_z^2 \mathbf{s}_\perp$$
$$= -\frac{1}{\tau_{ex}} \mathbf{s}_\perp \times \mathbf{e}_{\mathrm{M}_\parallel} - \frac{\mathbf{s}_\parallel}{\tau_{sf}} - \frac{\mathbf{s}_\perp}{\tau_{sf}}. \tag{4}$$

As suggested by *first principle* calculation [5] the amplitude of the adiabatic spin density $s_\parallel$ is frequency-insensitive and thus $\partial s_\parallel/\partial t$ is disregarded here. In the FMR dynamics, the transverse deviation $\mathbf{s}_\perp$ is found to be mainly dominated by the time variation of the magnetization $\mathbf{M}$, [3] the contribution of $\nabla_z^2 \mathbf{s}_\perp$ to the spin dynamics can be ignored, as well. $\partial \mathbf{s}_\perp/\partial t$ is on the order of $\partial^2 \mathbf{M}/\partial t^2$ and can be omitted in the linear response approximation. Under the above approximations, we obtain thus a closed form for the spin-density dynamics,

$$D_0 \nabla_z^2 s_\parallel = \frac{s_\parallel}{\tau_{sf}}, \tag{5}$$
$$s_\parallel \frac{\partial \mathbf{e}_{\mathrm{M}_\parallel}}{\partial t} = -\frac{1}{\tau_{ex}} \mathbf{s}_\perp \times \mathbf{e}_{\mathrm{M}_\parallel} - \frac{\mathbf{s}_\perp}{\tau_{sf}}. \tag{6}$$

The diffusion equation (5) results in an exponentially decaying surface spin density, $s_\parallel = C_\parallel e^{-z/\lambda_m}$ with $\lambda_m = \sqrt{D_0 \tau_{sf}}$ being the effective spin-diffusion length at the surface. $C_\parallel$ is determined by the electrical neutrality constraint, $C_\parallel = \eta \mathcal{P}_s/\lambda_m$, where $\mathcal{P}_s$ is the surface electron density due to the electrostatic screening. Clearly, $\mathbf{s}_\parallel$ can penetrate into the FM system within the spin diffusion length λ_m, which is over tens of nanometers in typical transition metals and alloys[6], giving rise to a marked interfacial ME interaction on overall thin FM films via the *s*-*d* exchange interaction,

$$F_{\mathrm{ME}} = \frac{1}{V} \int d\mathbf{r} J_{ex} \mathbf{s} \cdot \mathbf{e}_{\mathrm{M}_\parallel} = \frac{J_{ex}}{M_s} \bar{\mathbf{s}}_\parallel \cdot \mathbf{M} \tag{7}$$

where $\bar{s}_\parallel = \eta \mathcal{P}_s / d_{\mathrm{FM}}$ and d_{FM} is the FM film thickness. A contribution to the effective magnetic field is then given by $\mathbf{H}_{\mathrm{eff}}^{\mathrm{ME}} = -\delta F_{\mathrm{ME}}/\delta \mathbf{M}$ as

$$\mathbf{H}_{\mathrm{eff}}^{\mathrm{ME}} = -\frac{J_{ex}}{M_s} \bar{s}_\parallel \mathbf{e}_{\mathrm{M}_\parallel}. \tag{8}$$

Considering that

$$e\mathcal{P}_s = \epsilon E \tag{9}$$

with e and ϵ being the electron charge and the dielectric permittivity at the interface, respectively, the induced magnetization $\bar{s}_\parallel$ is linearly determined by the applied electric field E, so does the effective magnetic field.

The dynamic equation of $\mathbf{s}_\perp$, Eq. (6) deduces that

$$\mathbf{s}_\perp = -\frac{\tau_{ex}}{1+\xi^2}\left[\frac{\xi s_\parallel}{M_s}\frac{\partial \mathbf{M}}{\partial t} + \frac{s_\parallel}{M_s^2}\mathbf{M}\times\frac{\partial \mathbf{M}}{\partial t}\right] \tag{10}$$

with $\xi = \tau_{ex}/\tau_{sf}$. $\mathbf{s}_\perp$ in turn exerts a spin torque on the magnetization,

$$\begin{aligned}\mathbf{T}_{\mathrm{ME}} &= -\frac{J_{ex}}{\hbar M_s}\mathbf{M}\times\mathbf{s} \\ &= \frac{1}{1+\xi^2}\left[-\frac{s_\parallel}{M_s}\frac{\partial \mathbf{M}}{\partial t} + \frac{\xi s_\parallel}{M_s^2}\mathbf{M}\times\frac{\partial \mathbf{M}}{\partial t}\right].\end{aligned} \tag{11}$$

Comparing the functional structure of ME torque $\mathbf{T}_{\mathrm{ME}}$ with the terms appearing in the LLG equation, $\frac{\partial \mathbf{M}}{\partial t} = -\gamma\left(\mathbf{M}\times\mathbf{H}_{\mathrm{eff}}\right) + \frac{\alpha}{M_s}\left(\mathbf{M}\times\frac{\partial \mathbf{M}}{\partial t}\right)$, we conclude that when taking this ME torque into account it effectively renormalizes the LLG equation as

$$\tilde{\gamma} = \gamma/(1+\beta), \quad \tilde{\alpha} = (\alpha+\xi\beta)/(1+\beta) \tag{12}$$

with $\beta = \frac{s_\parallel}{M_s}\frac{1}{1+\xi^2}$. This makes evident that in general ME changes the effective magnetic field *and* the precessional damping, allowing to vary both by electric means, in as much as ME coupling is tunable electrically.

Especially, in the event that $\xi \ll 1$ and $\bar{s}_\parallel/M_s \ll 1$, e.g., in typical transition metal layer, one infers that $\tilde{\gamma} \approx \gamma$ and

$$\tilde{\alpha} = \alpha + \xi\frac{\bar{s}_\parallel}{M_s}, \tag{13}$$

where the averaged $\bar{s}_\parallel$ has been exploited to derive the effective damping constant $\tilde{\alpha}$. It should be noted that, as $\bar{s}_\parallel$ becomes increasingly negative by tuning the applied gate-voltage, it results in a linear growth of the effective magnetic field $H_{\mathrm{eff}}^{\mathrm{ME}}$ according to equation (8). By the same token we expect a decrease of the effective damping $\tilde{\alpha}$ given by equation (13), and even a *positive-to-negative transition* in $\tilde{\alpha}$ for sufficiently small intrinsic damping α at the critical point $E_c = (\alpha M_s e d_{\mathrm{FM}}) / (\xi\eta\epsilon\mu_B)$.

STATIC MAGNETOELECTRIC RESPONSE

The static magnetic properties of CoZr(20 nm)/PMN-PT response to external electric field are demonstrated in Fig.s1. M_s is not changed obviously when applying the electric field, which means that the change of the remnant magnetization M_r is caused by the induced magnetic anisotropy via the interfacial magnetoelectric effect.

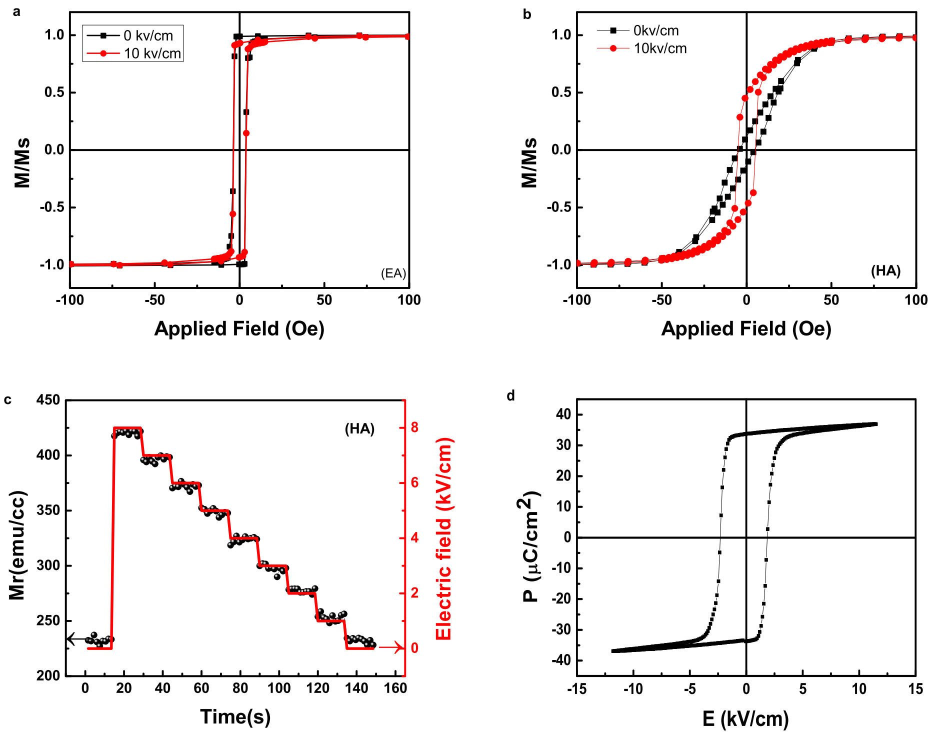

FIG. s1. The in-plane easy (a) and hard (b) axis magnetic hysteresis loops of CoZr(20 nm)/PMN-PT thin film under different electric field. The saturation magnetization M_s = 920 emu/cc. (c) The applied electric field and the changes of remnant magnetization M_r as a function of time. (d) Ferroelectric hysteresis loop of the structure.

RE-CALCULATION OF THE FMR ABSORPTION LINE SHAPE

FMR (or ESR) describes the resonant absorption of microwaves that match the effective Zeeman splitting of the energy levels in a FM material. Assuming an equilibrium magnetization $\mathbf{M} = [M_s, 0, 0]$, then for small cone angles of the precession the dynamic magnetization can be written as

$$\mathbf{M}(t) = \mathbf{M}_s + \mathbf{m}(t) \tag{14}$$

where $\mathbf{m}(t)$ is the rf component of $\mathbf{M}$ driven by the applied rf microwave field $\mathbf{h} = [0, 0, h]$. The dynamic components $\mathbf{m} = [0, m_y, m_z]$ has a time variation $e^{-i\omega t}$ and its magnitude is

much smaller than M_s. For low excitation rf field h, a linearization of the LLG equation

$$\frac{\partial \mathbf{M}}{\partial t} = -\gamma \left(\mathbf{M} \times \mathbf{H}_{\text{eff}} \right) + \frac{\alpha}{M_s} \left(\mathbf{M} \times \frac{\partial \mathbf{M}}{\partial t} \right) \tag{15}$$

leads two coupled equations,

$$-i\tfrac{\omega}{\gamma} m_y = h M_s - H_{\text{eff}} m_z + i\alpha \tfrac{\omega}{\gamma} m_z, \tag{16}$$

$$-i\tfrac{\omega}{\gamma} m_z = H_{\text{eff}} m_y - i\alpha \tfrac{\omega}{\gamma} m_y, \tag{17}$$

which yields the dynamic magnetic susceptibility χ_z:

$$\chi_z = \frac{m_z}{h} = \frac{M_s \left(H_{\text{eff}} - i\alpha \frac{\omega}{\gamma} \right)}{\left(H_{\text{eff}} - i\alpha \frac{\omega}{\gamma} \right)^2 - \left(\frac{\omega}{\gamma} \right)^2}. \tag{18}$$

The measured microwave power, $P(H)$, absorbed by a magnetic film is given by [7]

$$P(H) = \frac{1}{2} \omega \Im \chi_z |h|^2. \tag{19}$$

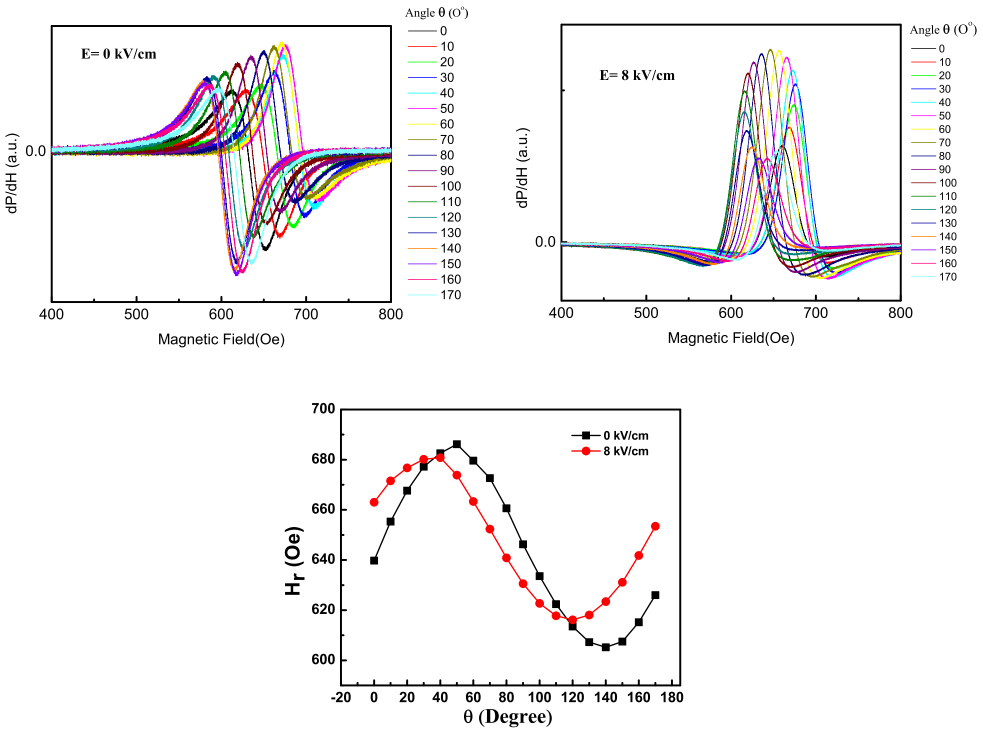

FIG. s2. Angular-dependence of FMR spectra of CoZr(20nm)/PMN-PT with $E = 0$ kV/cm and $E = 8$ kV/cm when rotating the static magnetic field in the plane under different external electric fields E. H_r are modeled well by Kittle formula with an in-plane uniaxial magnetic anisotropy.

FMR therefore occurs when the imaginary part of the magnetic susceptibility $\Im\chi_y$ is maximal. For small damping one can set $\alpha \approx 0$ the maximum is given then by (Kittel formula)

$$\frac{\omega}{\gamma} = H_{\text{eff}}. \tag{20}$$

Under certain the conditions FMR spectra are found to have almost perfect Lorentzian shape[8]. This can be shown by replacing H_{eff} with $H_r + \delta H$. In linear $\delta H - i\alpha\frac{\omega}{\gamma}$ the dynamic susceptibility becomes

$$\chi_z = \frac{M_s}{2H_{\text{r}}} \frac{H_{\text{r}} + \delta H - i\alpha\frac{\omega}{\gamma}}{\delta H - i\alpha\frac{\omega}{\gamma}}. \tag{21}$$

Rationalizing the denominator yields the imaginary and the real parts of χ_z,

$$\Im\chi_z = \frac{M_s}{2}\frac{\Delta}{\Delta^2 + (H_{\text{eff}} - H_r)^2}, \tag{22}$$

$$\Re\chi_z = \frac{M_s}{2H_{\text{r}}} + \frac{M_s}{2}\frac{H_{\text{eff}} - H_{\text{r}}}{\Delta^2 + (H_{\text{eff}} - H_{\text{r}})^2}. \tag{23}$$

Considering the profile, the imaginary part $\Im\chi_z$ represents a symmetric Lorentzian with a half-width at half-maximum (HWHM) $\Delta = \alpha\frac{\omega}{\gamma}$. Whereas, the real part $\Re\chi_z$ is antisymmetric and has a zero-crossing point at $H_{\text{eff}} = H_r$. It should be noted that, in general, the magnetization is probed with a certain phase correlation with respect to the microwave excitation, the FMR spectrum does not correspond to only imaginary part of the susceptibility, but represents a mixture of imaginary and real parts [9]. Hence, the actual fit function can be given by an asymmetric Lorentzian function:

$$P(H) = A\,\frac{\Delta\cos\phi + (H_{\text{eff}} - H_r)\sin\phi}{\Delta^2 + (H_{\text{eff}} - H_r)^2} \tag{24}$$

where ϕ is the phase which mixes the real and imaginary parts of the dynamic susceptibility. $\Delta_{\text{eff}} = \Delta\cos\phi$ is the effective inhomogeneous line broadening of FMR spectra.

The angular dependence of FMR spectra of CoZr(20nm)/PMN-PT when varying the applied magnetic field in plane is presented in Fig. s2.

[1] Jia, C. L., Wei, T. L. , Jiang, C. J. , Xue, D. S., Sukhov, A. & Berakdar, J. Mechanism of interfacial magnetoelectric coupling in composite multiferroics. *Phys. Rev. B* **90**, 054423 (2014)

[2] Tatara, G. , Kohno, H. & Shibata, J. Microscopic approach to current-driven domain wall dynamics. *Physics Reports* **468**, 213 (2008).

[3] Zhang, S. & Li, Z. Roles of nonequilibrium conduction electrons on the magnetization dynamics of ferromagnets. *Phys. Rev. Lett.* **93**, 127204 (2004).

[4] Soulen, R. J. *et al.* Measuring the spin polarization of a metal with a superconducting point contact. *Science* **282**, 85 (1998).

[5] Rondinelli, J. M., Stengel, M. & Spaldin, N. A. Carrier-mediated magnetoelectricity in complex oxide heterostructures. *Nature Nanotech* **3**, 46 (2007)

[6] Bass, J. & Jr. Pratt, W. P. Spin-diffusion lengths in metals and alloys, and spin-flipping at metal/metal interfaces: an experimentalists critical review. *J. Phys.: Condens. Matter* **19**, 183201 (2007).

[7] Yalcin O. (Ed.): Ferromagnetic Resonance - Theory and Applications; DOI:10.5772/50583 (InTech, **2013**)

[8] Celinski, Z., Urquhart, K. B. & Heinrich, B. Using ferromagnetic resonance to measure the magnetic moments of ultrathin films. *Journal of Magnetism and Magnetic Materials* **166**, 6 (1997).

[9] Hoffmann, F. *et al.* Mapping the magnetic anisotropy in (Ga,Mn)As nanostructures. *Phys. Rev. B* **80**, 054417 (2009).
